# Fishmeal Protein Replacement by Defatted and Full-Fat Black Soldier Fly Larvae Meal in Juvenile Turbot Diet: Effects on the Growth Performance and Intestinal Microbiota

**DOI:** 10.1155/2023/8128141

**Published:** 2023-04-12

**Authors:** Jingjing Zhao, Jintao Pan, Zhonghao Zhang, Zhichu Chen, Kangsen Mai, Yanjiao Zhang

**Affiliations:** ^1^Key Laboratory of Aquaculture Nutrition and Feed, Ministry of Agriculture, The Key Laboratory of Mariculture, Ministry of Education, Ocean University of China, 5 Yushan Road, Qingdao 266003, China; ^2^Qingdao National Laboratory for Marine Science and Technology, 1 Wenhai Road, Qingdao 266237, China

## Abstract

A 12-week feeding trial was conducted to investigate the effect of the same fishmeal protein level replaced by black soldier fly larvae (*Hermetia illucens*) meal (BSFL) with different lipid contents on the growth performance and intestinal health of juvenile turbot (*Scophthalmus maximus* L.) (initial body weight 12.64 g). Three isonitrogenous and isolipidic diets were formulated: fish meal-based diet (FM), diets DF and FF, in which 14% fish meal protein of the FM diet was replaced by defatted and full-fat BSFL, respectively. There were no significant differences in growth performance, intestinal morphology, and mucosal barrier function between the DF and the FM group. However, diet FF markedly reduced the growth performance, intestinal perimeter ratio, and the gene expression of anti-inflammatory cytokine *TGF-β* (*P* < 0.05). Compared to group FF, the communities of intestinal microbiota in group DF were more similar to group FM. Moreover, diet DF decreased the abundance of some potential pathogenic bacteria and enriched the potential probiotics, such as *Bacillus*. Diet FF obviously altered the composition of intestinal microbiota and increased the abundance of some potential pathogenic bacteria. These results suggested that the application of defatted BSFL showed more positive effects on fish growth and intestinal health than the full-fat BSFL, and the intestinal microbiota was closely involved in these effects.

## 1. Introduction

Recently, more attention has been paid to insect meal since insects serve as an important part of wild fish food. More importantly, insects are economical and highly available feed ingredients with a high protein content (30%-65%) [1, 2]. Black soldier fly larvae (*Hermetia illucens* L., BSFL), as a saprophytic insect, can feed on livestock manure and household waste. BSFL meal has been widely used in feeds of livestocks, poultry, and aquaculture due to its rapid reproduction, large biomass, high absorption rate, good palatability, and easy management [3, 4].

The lipid content of BSFL meal products varies greatly due to different processing techniques. In general, the lipid content of defatted BSFL meal is below 30% while that of full-fat BSFL meal is above 30% [5]. Previous studies have shown that when used in diets, BSFL meal with different lipid contents differently affected the growth performance and health of fish. However, limited information has been available about the comparison of the application efficacy of BSFL with different lipid contents in fish feeds. Most previous studies showed that a diet with appropriate defatted BSFL meal supplementation positively affected the growth performance or physiological response of fish. For example, diets with 5.3%-40% defatted BSFL meal (lipid content: 8%-25%) did not affect the growth performance, digestive enzyme activity, and the liver and distal intestine morphology in European seabass (*Dicentrarchus labrax*), Jian carp (*Cyprinus carpio* var. Jian), Siberian sturgeon (*Acipenser baerii*), turbot (*Psetta maxima*), totoaba (*totoaba macdonaldi)*, rainbow trout (*Oncorhynchus mykiss*), and Eurasian perch (*Perca fluviatilis*) and even improved the antibacterial ability of barramundi (*Lates calcarifer*) [6–13]. Moreover, the high lipid content of BSFL meal was also an important alternative lipid source for fish feeds. However, conflicting results have been reported when full-fat BSFL meal were used in fish feeds. Negative effects on growth performance were observed in pearl gentian grouper (*Epinephelus fuscoguttatus ♀×Epinephelus lanceolatus ♂*) and Atlantic salmon (*Salmo salar*) [14, 15], whereas in other studies, dietary full-fat BSFL meal improved the growth performance of Thai climbing perch (*Anabas testudineus Bloch*) and Japanese seabass (*Lateolabrax japonicas*), as well as the quality of Jian carp [16–18]. In addition, the application of full-fat BSFL meal in largemouth black bass *(Micropterus salmoides)* significantly improved the antioxidant capacity and immunity of fish [19]. Therefore, a comparative study on BSFL with different lipid contents used in fish feed is of great significance.

Currently, most studies on insect meal application in fish feeds focus on fish growth and nutrient digestion. As well known, a healthy intestinal tract is crucial for the growth performance and health of fish. The intestinal, mechanical, and immunologic barriers are the primary line of defense against pathogen infection [20–23]. Moreover, a beneficial intestinal microbiota is essential for maintaining the health of the intestine and host. In recent years, numerous studies in fish have reported that the intestinal microbiota play basilic roles in the host development, physiological response, and metabolism [1, 24–27]. In general, the feed can shape the community of intestinal microbiota. Consequently, intestinal microbes can also affect a variety of physiological activities of the host, including nutrient digestion and absorption [1, 24, 28]. Therefore, it is crucial to evaluate the fish intestinal microbiota in response to insect ingredients.

Turbot (*Scophthalmus maximus* L.) is an important marine culture fish species in the world. Because it is a carnivorous fish species, it is particularly urgent to look for high-quality and low-price ingredients to replace fish meal and fish oil in the turbot diets. The purpose of this study was to comprehensively evaluate the effects of BSFL meal with different lipid contents on the growth and intestinal health of juvenile turbot. The results would provide useful information for the BSFL meal application in fish feeds.

## 2. Materials and Methods

### 2.1. Experimental Diets

Three isonitrogenous and isolipidic diets were formulated to contain about 52% protein and 12% lipid. The full-fat BSFL meal (the black soldier fly larvae were propagated and grown on kitchen waste) was provided by Guangdong Wuliang Biotechnology Co., Ltd. (Guangdong, China). The defatted BSFL meal was obtained by petroleum ether extraction in the laboratory (Soxtec™ 8000, FOSS, Sweden). The crude protein of defatted BSFL and full-fat BSFL were 43.45% and 31.37%, respectively, while the crude lipid of them was 23.77% and 43.21%, respectively. The main protein sources of the control diet (FM) were the fish meal, wheat meal, and soybean meal. Other two diets (DF and FF) were formulated, in which 14% fishmeal protein was replaced by defatted BSFL and full-fat BSFL, respectively (Table 1). The amino acid composition of the three diets and three raw materials is shown in Supplementary Table S1 and S2, and their fatty acid composition is shown in Tables 2 and 3.

The feed was made, packed, and stored according to the description of Chen et al. [29]. Firstly, the solid feed materials were thoroughly crushed through an 80-mesh screen and evenly step-by-step mixed according to the feed formula. Secondly, fish oil and soy lecithin were added and thoroughly mixed. Finally, about 30% distilled water was added and mixed thoroughly. The pellet feed with a diameter of 3 mm was prepared by Bait granulating machine (version: EL, Huatong Printing and Dyeing Machinery Co., Ltd., China), dried for 12 h at 55°C, and then stored at -20°C.

### 2.2. Experimental Fish and Feeding

The juvenile turbots were purchased from Shandong Kehe Marine High Technology Co., Ltd. (Weihai, China). Before the experiment, turbots were fed with commercial feed (Shandong Kehe Marine High Technology Co., Ltd., Weihai, China) for 2 weeks to acclimate to the experimental condition. A total of 360 fish with uniform size (initial body weight 12.64 g) were randomly assigned to 9 glass fiber tanks (200 L). The whole experiment was implemented in an indoor flow-through water system in Yellow Sea Fisheries Co., Ltd. (Haiyang, China). The fish were fed to apparent satiation at 6 : 30 am and 6 : 30 pm every day for 12 weeks. The fish in each tank were fed to apparent satiation. After feeding, uneaten feeds were collected 15 minutes later and their weight was calculated by getting an average weight of each pellet. Finally, the accumulated feed intake of fish in 12 weeks was calculated to carry out the statistical analysis of FI. During the feeding trial, dissolved oxygen was higher than 7 mg/L; water temperature ranged from 16°C to 21°C; pH 7.59-7.84; and salinity 28-30.

### 2.3. Sample Collection

All fish in each tank were weighed and counted when the feeding experiment terminated. The turbots were anesthetized with eugenol before sampling. The distal intestine of three randomly selected fish from each tank was collected and then fixed in Bouin's fluid (RS4141, G-CLONE, China) for distal intestine histological analysis. The distal intestine of two randomly selected fish from each tank was cut with a sterile dissector around the alcohol lamp for intestinal microflora analysis. The distal intestines were transferred to 1.8 ml sterile tubes (Nunc, America), immediately put into liquid nitrogen, and then stored at -80°C. All fish-rearing practices and sampling protocols in this study were approved by the Animal Care and Use Committee of the Ocean University of China.

### 2.4. Intestinal Histology

After 24 hours of fixation in Bouin's fluid, the intestine was dehydrated and then made into paraffin blocks. Sections of 7 *μ*m were cut (MX35 Ultra Microtome Blade, Thermo Scientific, USA) and stained with haematoxylin-eosin (HE) (BC-DL-001, SenBeiJia Biological Technology Co., Ltd., China). The slides were observed by a light microscope (DP72, Olympus, Japan) to acquire images. The micrographs were analyzed by Image-Pro Plus (version 6.0.0.260 S/N 41 M60032-00032, USA). The perimeter ratio (PR) was determined according to the description of Dimitroglou et al. [30]. The PR of the intestine was the ratio of the internal perimeter (IP) to the intestine lumen and the external perimeter (EP) of the intestine (PR = IP/EP).

### 2.5. Real-Time Quantitative PCR

Total RNA was extracted from distal intestines using RNAex Pro Reagent (Accurate Biotechnology Co., Ltd., Hunan, China). The RNA concentration was instrumented by NanoDrop®2000. The RNA was reversed transcribed to cDNA with *Evo M-MLV* reverse transcription premix kit.

The specific primers of genes were synthesized by Sangon (Shanghai, China) (Table 4). The housekeeping gene was *β-*actin. The qPCR assays were carried out in a quantitative thermal cycler (Bio-Rad, USA) The reaction volume was 20 *μ*L containing 10 *μ*L SYBR® Green Premix Pro Taq HS qPCR Kit (AG11701, Accurate Biotechnology (Hunan) Co., Ltd., China), 6.4 *μ*L RNase free water, 0.8 *μ*L primer F (10 *μ*M), 0.8 *μ*L primer R (10 *μ*M), and 2 *μ*L of DNA template (<100 ng). The real-time PCR amplification program started with 1 min at 95°C, followed by 40 cycles “5 s at 95°C, 30 s at 60°C.” The expression levels of these genes were calculated by the 2^−ΔΔCT^ method [31].

### 2.6. Intestinal Microbiota Profiling

The total genome DNA of distal intestine samples was extracted, amplified, and sequenced by Novogene (Beijing, China). In order to analyze the diversity and composition of the intestinal bacterial community, the V4 region of the 16S rRNA gene of intestinal bacteria was amplified with 515f/806r primer after extraction, using the Illumina NovaSeq platform to sequence.

After the deletion of barcode and primer sequences and splicing by FLASH, the raw tags were obtained. The high-quality cleaning tags were obtained after strict filtering of raw tags [32]. The effective tags were obtained by comparison with the species annotation database and by removing the chimeric sequences [33]. All effective tags of all samples were clustered using the UPARSE software, and Operational Taxonomic Units (OTUs) with 97% consistency were the sequence [34]. Species annotation analysis was performed with the Mothur method and SSUrRNA database of SILVA138 (threshold set at 0.8~1) to obtain taxonomic information [35, 36]. The QIIME software was used to calculate the alpha diversity index (observed species, chao1, ace, Shannon index, and Simpson index) and the unweighted UniFrac distance of beta diversity. Rarefaction curve, PCoA plots, and UPGMA clustering were plotted using the R software. Metastats analysis was performed to identify bacterial taxa with differences between different treatments at the genus level [37].

### 2.7. Calculations

The calculation formula was as follows:

(1) $$\begin{array}{c} \text{Specific growth rate }\left(\text{SGR},\%/\text{day}\right)=100\times \frac{\ln\;W_{F}–\ln\;W_{I}}{t},\\ \text{Weight gain }\left(WG,\%\right)=100\times \frac{W_{F}–W_{I}}{W_{I}},\\ \text{Feed efficiency }\left(FE\right)=\frac{W_{F}–W_{I}}{D},\\ \text{Feed intake }\left(FI,\%/\text{day}\right)=\frac{100\times D}{\left[\left(W_{F}+W_{I}\right)/2\right]/t}\\ \text{Survival }\left(\%\right)=100\times \frac{A_{F}}{A_{I}}, \end{array}$$

where *W*_*F*_ and *W*_*I*_ are the final and initial fish weight, respectively; *t* is the days of feeding duration; *D* is the total feed intake which is calculated on a dry matter basis; and *A*_*I*_ and *A*_*F*_ are the initial and final fish number, respectively.

### 2.8. Statistical Analysis

The data analysis software was IBM SPSS Statistics 22. One-way analysis of variance and Tukey's multiple comparison were used to test the significance of the difference between the mean values. Final results were presented as means with standard error (mean ± SEM). *P* < 0.05 was regarded as a significant difference between groups.

## 3. Results

### 3.1. Growth Performance

The FBW, SGR, WG, and FE of turbot were significantly lower in the FF group than those in the FM group (*P* < 0.05). However, no significant difference in these results was observed between the FM and DF groups (*P* > 0.05) (Table 5).

### 3.2. Intestinal Histology

**Figure 1:**
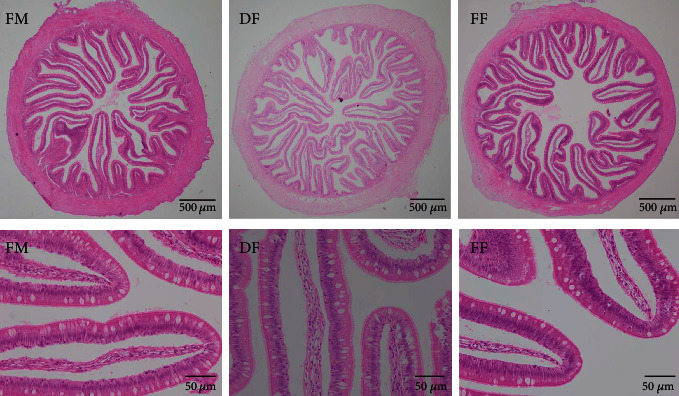
Effects of dietary different lipid content BSFL meal on the distal intestinal morphology in turbot. H&E staining. The intestinal tissue of all treatment groups was well formed without inflammatory cell infiltration.

**Figure 2:**
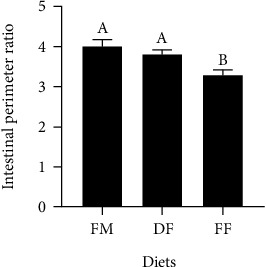
The intestinal perimeter ratio in turbot. The different letters on the bar chart represent significant differences in the mean values (*n* = 6) between the groups (*P* < 0.05).

No obvious changes were observed in the distal intestine of turbot from all treatments (Figure 1). However, the lowest PR of the distal intestine was observed in turbot-fed diet FF (Figure 2).

### 3.3. Intestinal Microbiota

**Figure 3:**
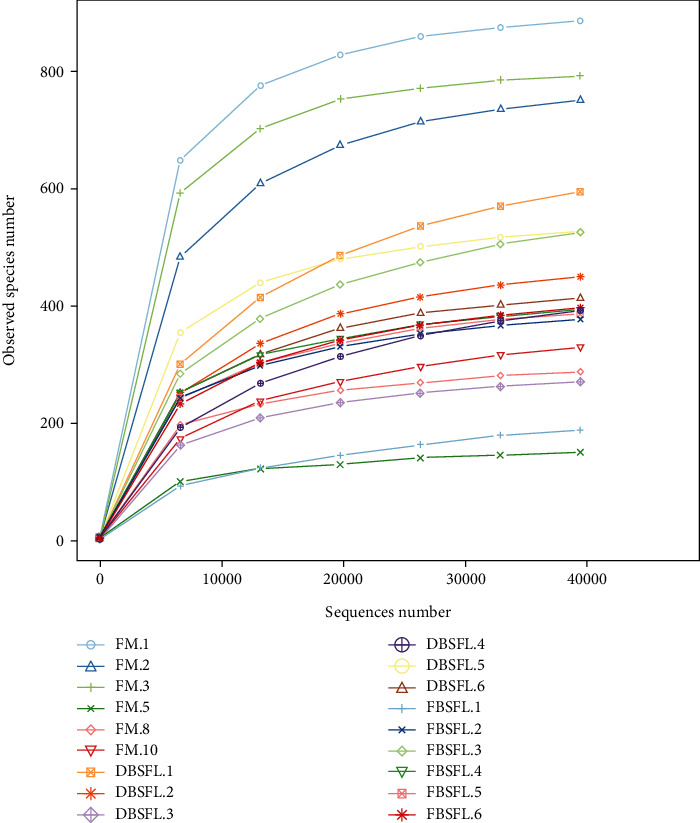
Rarefaction curve of intestinal microbiota on turbot. Abbreviations: FM.1, FM.2, FM.3, FM.5, FM.8, and FM.10 were the six samples of the FM group; DBSFL.1, DBSFL.2, DBSFL.3, DBSFL.4, DBSFL.5, and DBSFL.6 were the six samples of the DF group; FBSFL.1, FBSFL.2, FBSFL.3, FBSFL.4, FBSFL.5, and FBSFL.6 were the six samples of the FF group.

A total of 1,460,860 raw tags were obtained by high-throughput sequencing. After filtration, 1,452,459 clean tags were obtained. After merging, 1,292,666 tags were obtained from 18 samples, clustering into 23,608 OTUs with over 97% sequence similarity in total. The rarefaction curve showed that all samples had reached the saturation stage, indicating that the sequencing depth was sufficient and the data was reliable (Figure 3).

**Figure 4:**
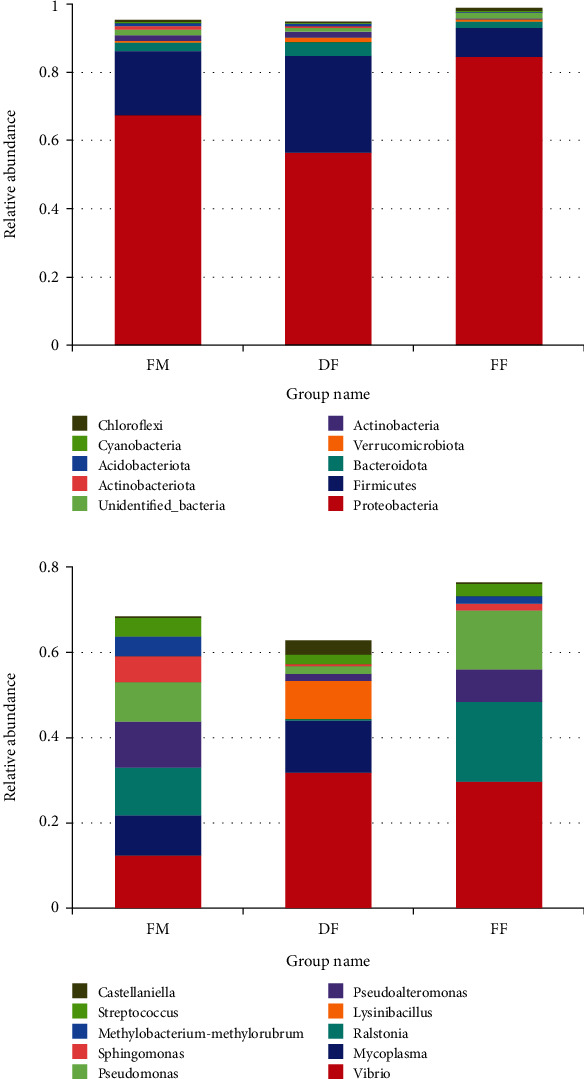
Taxonomic levels of the top ten phyla (a) and genera (b) in relative abundance.

At the phylum, Proteobacteria and Firmicutes were the predominant bacterial phyla in the intestine of turbot in this study (Figure 4(a)). At the genus level, the dominant bacteria in group FM were *Vibrio* (12.56%), *Mycoplasma* (9.24%), *Ralstonia* (11.33%), *Pseudoalteromonas* (10.67%), and *Pseudomonas* (9.33%); the dominant bacteria in group DF were *Vibrio* (31.84%), *Mycoplasma* (12.31%), and *Lysinibacillus* (9.08%); the dominant bacteria in group FF were *Vibrio* (29.70%), *Ralstonia* (18.78%), *Pseudoalteromonas* (7.59%), and *Pseudomona* (13.83%) (Figure 4(b)). The alpha diversity results showed that there was no significant difference in diversity and richness among all treatments (*P* > 0.05) (Table 6).

**Figure 5:**
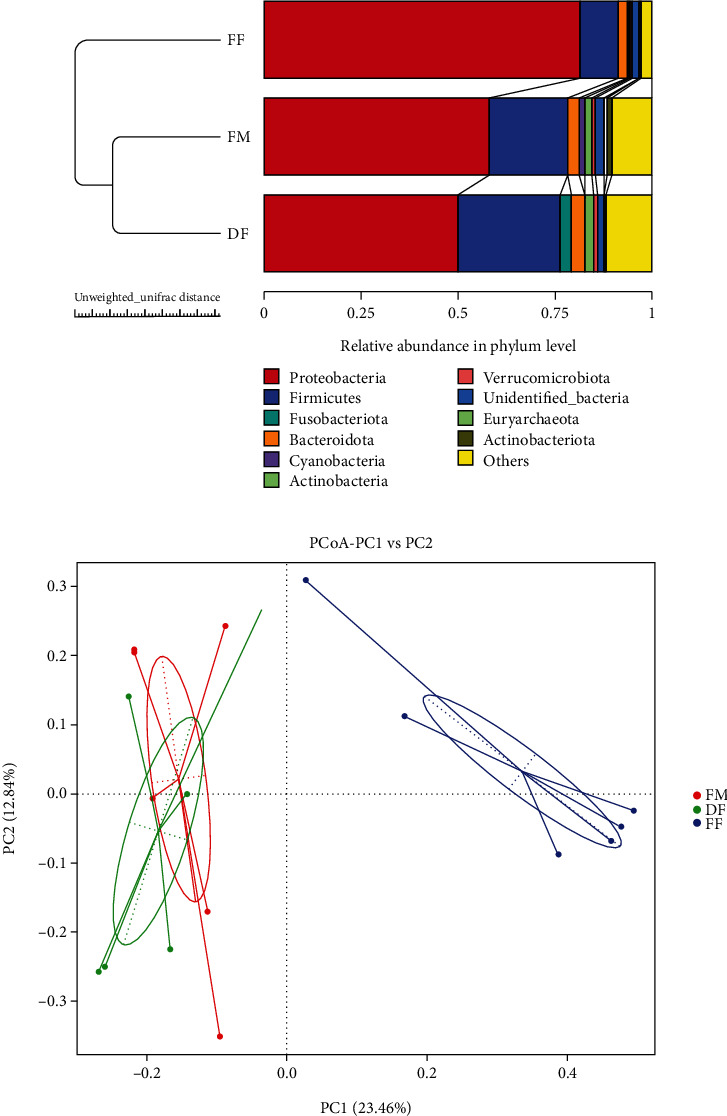
Beta diversity of intestinal microbiota of turbot. (a) Group UPGMA clustering tree. (b) PCoA plot.

The PCoA results showed that the distance between the FM and DF groups was shorter, and both of them were far from the FF group. This was consistent with the UPGMA clustering tree results (Figure 5).

**Figure 6:**
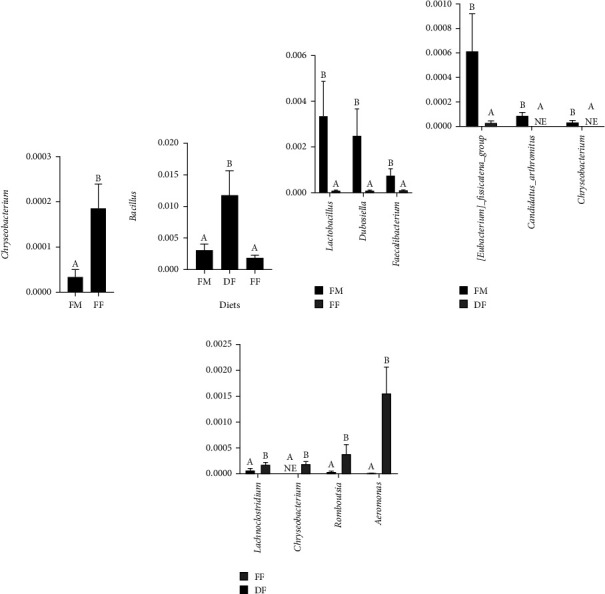
Metastat analysis on the distal intestine of turbot at the genus level. The different letters on the bar chart represent significant differences in the mean values (*n* = 6) between the groups (*P* < 0.05).

The Metastat analysis showed that the relative abundance of *Candidatus_Arthromitus*, *Chryseobacterium*, and *[Eubacterium]_fissicatena_group* was significantly decreased (*P* < 0.05) in the DF diet compared with the FM diet (Figure 6(d)). However, the abundance of *Bacillus* was significantly higher (*P* < 0.05) in the fish-fed DF diet compared with group FF or FM (Figure 6(b)). The relative abundance of *Lactobacillus, Dubosiella,* and *Bifidobacterium* was significantly lower (*P* < 0.05) in the FF group than those in the FM group (Figure 6(c)). The relative abundance of *Chryseobacterium* was significantly higher (*P* < 0.05) in the FF group than those in the FM group (Figure 6(a)). Compared with diet DF, diet FF remarkably (*P* < 0.05) increased the relative abundance of *Lachnoclostridium*, *Romboutsia*, *Aeromonas,* and *Chryseobacterium* (Figure 6(e)).

### 3.4. Intestinal Mucosal Barrier Function

**Figure 7:**
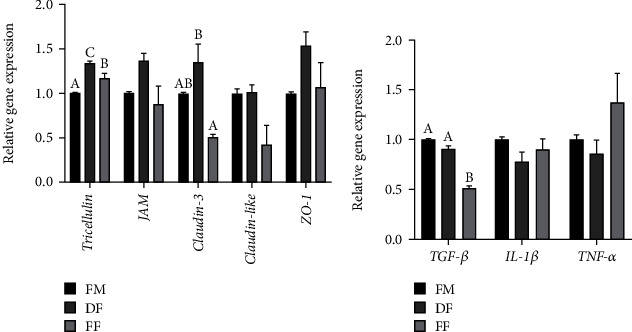
The gene expression of mechanical barrier (a) and immunologic barrier (b) in distal intestine of experimental turbot. The different letters on the bar chart represent significant differences in the mean values (*n* = 6) between the groups (*P* < 0.05).

Compared to the FM diet, the DF and FF diets significantly increased the expression of *Tricellulin* (*P* < 0.05). The expression of *Claudin-3* was significantly upregulated in the turbot-fed DF diet than those fed the FF diet (*P* < 0.05) (Figure 7(a)). The FF diet significantly decreased the gene expression of *TGF-β* (*P* < 0.05). No significant differences in the expression of *IL-1β* and *TNF-α* were observed among the three groups (*P* > 0.05) (Figure 7(b)).

## 4. Discussion

In this study, fish meal replacement by black soldier fly larvae (BSFL) meal with different lipid content had different effects on the growth performance of turbot. Dietary defatted BSFL (DF) did not affect growth performance, which was consistent with previous studies on large yellow croakers, Eurasian perch, Japanese seabass, Nile tilapia, and Jian carp [10, 12, 38–40]. However, dietary full-fat BSFL (FF) significantly reduced the growth performance (SGR, WG, and FE) of turbot. The effects of full-fat BSFL application in fish have been controversial among different studies. Dietary full-fat BSFL meal at 30% had positive effects on the growth performance of sturgeons [41]. However, a diet with 15% full-fat BSFL supplementation suppressed the growth performance (FCR and WG) of Atlantic salmon and pearl gentian grouper [14, 42]. The lower feed efficiency of diet FF might contribute to the differences in the present study. Moreover, fish-fed diet FF had a lower perimeter ratio of the intestine. In general, the lower the PR value, the smaller the intestinal absorption area is, which represents the worse digestion and absorption capacity of fish [30]. Based on the BSFL meal level in experimental diets and the reported chitin content in BSFL, it can be calculated that the content of chitin in diet DF is about 1.16% and that in diet FF is about 0.59% [9, 43]. The presence of chitin could reduce the growth performance of fish [15, 42, 44, 45]. Besides, Kroeckel et al. [9] had shown that chitin-degrading enzymes did not exist in the intestine of turbot. Besides, there was no additional fish oil in diet FF because of the high lipid content of full-fat BSFL meal. Therefore, the contents of DHA and EPA in diet FF are significantly lower than those in diet DF. According to previous studies, insufficient DHA and EPA could impair the development of fish [46–49]. In addition, it has been reported that n-3 long-chain-polyunsaturated fatty acids (mainly DHA and EPA) could enhance intestinal immune functions and positively shape the host microbial ecosystem [50, 51]. The DHA and EPA contents in the DF group were higher compared to the FF group in this study.

The intestinal microecology is a complex and dynamic ecosystem and intestinal microbiota can play various functions in hosts [27, 52–54]. At the phylum level, Firmicutes and Proteobacteria were the dominant bacteria in all groups, which was consistent with previous studies in turbot [55–58]. At the genus level, *Vibrio*, *Mycoplasma*, *Ralstonia*, *Pseudoalteromonas*, *Lysinibacillus*, and *Pseudomonas* were the dominant microbiota in this study. *Vibrio* is a genus of ubiquitous heterotrophic bacteria in aquatic environments [59]. Some species are opportunistic pathogens and some of them also can act as potential probiotics to improve the intestinal digestion function of turbot and disease resistance of sturgeon [60, 61]. *Mycoplasma* has been known as a pathogen, but recently, it has also been shown that the abundance of *Mycoplasma* in the intestine was negatively correlated with the abundance of potentially pathogenic bacteria in trout and Atlantic salmon [62, 63]. Besides, the *Lysinibacillus* facilitated the degradation of protein-like substances [64]. Species of *Pseudoalteromonas* are used as probiotics in aquaculture for their antibacterial, bacteriolytic, and algicidal activities [65–68]. *Ralstonia* and *Pseudomonas* were usually regarded as an opportunistic pathogenic bacterium [69–71]. The results of the TOP genus showed a higher abundance of *Vibrio*, *Mycoplasma*, and *Lysinibacillus* in fish-fed diet DF. In addition, the PCoA and UPGMA plot results showed that the distance between group FM and DF was short, but both of them were far from group FF. Collectively, these results indicate that diet DF has a more positive effect on the intestinal microbiota composition than diet FF.

According to the MetaStat analysis, the abundance of *Bacillus* was significantly increased in fish-fed diet DF. Similarly, the abundance of *Bacillaceae* (including *Bacillus*) was higher in rainbow trout-fed diets with 30% BSFL meal, especially defatted BSFL meal [72]. In this study, the higher content of DHA and EPA in diet DF than that in diet FF might be responsible for variation in *Bacillus* abundance. In mice, dietary EPA or DHA enriched the beneficial bacteria such as *Lactobacillus* in the intestine [73, 74]. As known, insects contain chitin, which could affect the digestion capacity and growth performance of fish [9, 75, 76]. Studies have shown that *Bacillus* could produce chitin-degrading enzymes in the intestine of Atlantic salmon and rainbow trout [72, 77, 78]. In this study, a higher abundance of *Bacillus* in fish-fed diet DF may reduce the chitin content to some extent, ultimately improving feed efficiency and growth of turbot. Besides, the relative abundance of *Candidatus_Arthromitus*, *Chryseobacterium*, and *[Eubacterium]_fissicatena_group* was lower in the DF group than that in the FM group. These bacteria were frequently considered as a pathogenic bacterium [79–83]. However, the relative abundance of potential probiotics *Lactobacillus*, *Dubosiella*, and *Bifidobacterium* [84–86] was decreased in the FF group, and the potential pathogen *Chryseobacterium* [81] was increased in this group. *Dubosiella* was considered a potential probiotic because it is positively correlated with butyric acid levels [87]. *Bifidobacterium* is butyrate-producing bacteria, and the increased short-chain fatty acids were crucial to the improvement of immunity in hosts [88–91]. In the present study, diet FF also increased the abundance of pathogenic bacterium *Lachnoclostridium*, *Romboutsia*, *Chryseobacterium*, and *Aeromonas*. *Lachnoclostridium* can cause metabolic disorders in mice [92]. *Romboutsia* was the main reason for diarrheal shellfish poisoning events in human beings over the world [93]. *Aeromonas* can easily lead to deadly and contagious diseases for marine fish [94]. Overall, the intestinal microbiota of turbot is sensitive to the lipid content and other dietary compositions and may play key roles in the utilization efficiency of BSFL meal by a fish host. Although this experiment can illustrate the changes of the intestinal microbiota of turbot by replacing fish meal with defatted and full-fat BSFL, it would be better if there are more intestine samples used. Future relevant studies may use a bigger sample number.

In this study, both the DF and FF diets upregulated the gene expression of *Tricellulin*, while diet FF downregulated the *Claudin-3* expression. The expression of *TGF-β* was also significantly downregulated by diet FF. As well known, tight junction proteins are important parts of the intestinal mechanical barrier [95], and *TGF-β* plays a vital role in the control of immune homeostasis and prevention of intestinal inflammation [96, 97]. The present results suggested that dietary full-fat BSFL meal has a negative effect on the intestinal mucosal barrier.

## 5. Conclusions

In the case of replacing the same fishmeal protein level, the BSFL meal with different lipid contents had significantly different effects on the growth performance and intestinal health of turbot. The BSFL with lower lipid content (23.77%) had no adverse effect on growth performance and increased the abundance of some potential probiotics including *Bacillus*. However, the BSFL with full-fat (43.21%) suppressed the growth performance and disrupted the balance of intestinal microbiota. More attention should be paid to the lipid content of BSFL meal and the changes of fatty acid composition in the diet when it is used in fish feeds. In particular, the roles of intestinal microbiota in the BSFL meal effects need to be further explored.

## Figures and Tables

**Table 1:** Formulation and proximate composition of the experimental diets (% dry matter).

Ingredients (%)	FM	DF	FF
Fish meal^a^	52.00	44.72	44.72
Defatted BSFL^b^	0.00	12.12	0.00
Full-fat BSFL^c^	0.00	0.00	16.36
Wheat meal^a^	19.30	16.36	16.42
Corn gluten meal^a^	10.00	10.00	10.00
Wheat gluten^a^	2.00	2.00	2.00
Soybean meal^a^	8.00	8.00	8.00
Fish oil^a^	5.20	3.30	0.00
Soybean lecithin	1.00	1.00	0.00
Vitamin premix^d^	1.00	1.00	1.00
Mineral premix^e^	0.50	0.50	0.50
Choline chloride	0.30	0.30	0.30
Monocalcium phosphate	0.50	0.50	0.50
Ethyoxyl	0.05	0.05	0.05
Y_2_O_3_	0.10	0.10	0.10
Calcium propionate	0.05	0.05	0.05
Total	100.00	100.00	100.00
Nutrient composition of feed			
Crude protein	52.27	51.72	51.79
Crude lipid	10.63	9.96	11.58

^a^Fish meal: CP 70.49%, CL 10.59%; wheat meal: CP 16.42%; corn gluten meal: CP 66.9%; gluten meal: CP 80.63% soybean meal: CP 49.32% (CP: crude protein, CL: crude lipid). ^b^Defatted BSFL meal: CP 43.45%, CL 23.77%. ^c^Full-fat BSFL meal: CP 31.37%, CL 43.21%. ^d^Vitamin premix (g·kg^−1^ diet): vitamin A ≥ 1,140,000 IU; vitamin D3 ≥ 180,000 IU; DL − *α* − tocopherol acetate ≥ 7.6 g; menadione acuity ≥ 1.2 g; thiamine nitrate ≥ 0.93 g; riboflavin acuity 1.35 g; pyridoxine hydrochloride ≥ 1.10 g; cyanocobalamin acuity ≥ 0.0075 g; calcium d − pantothenate ≥ 4.5 g; nicotinamide acuity ≥ 6.75 g; folic acid ≥ 0.465 g; D biotin ≥ 0.0475 g; L − ascorbate − 2 phosphate (calculated by L − ascorbate) ≥ 16.7 g; inositol ≥ 10 g; and moisture ≤ 10%. ^e^Mineral premix (g·kg^−1^ diet): Fe ≥ 37.14 g; Zn ≥ 16.43 g; Mn ≥ 3.03 g; Cu ≥ 0.95 g; Co ≥ 0.1 g; Se ≥ 0.04 g; I ≥ 0.1 g; moisture ≤ 10%.

**Table 2:** Fatty acid composition of three raw materials (% total fatty acids).

Fatty acid	Fish meal	Defatted BSFL	Full-fat BSFL
C12:0	0.33	16	15.43
C14:0	5.59	3.95	4.04
C15:0	0.57	0.12	0.12
C16:0	25.32	22.59	23.25
C17:0	1.58	0.22	0.22
C18:0	9.67	4.25	4.06
C20:0	2.8	0.23	0.18
C21:0	0.21	0.02	0.01
C22:0	0.71	0.11	0.06
C23:0	0.37	0	0
C24:0	1.79	0.07	0.05
*Σ*SFA	48.93	47.55	47.42
C15:1	0.44	0.14	0.1
C16:1	6.03	0.04	2.45
C17:1	1.08	0.03	0.2
C18:1trans	0.41	0.01	29.04
C18:1CIs	15.65	0.06	0.66
C22:1	0.15	0	0.04
*Σ*MUFA	23.76	0.28	32.49
C18:2n6t	0.07	0.03	0.04
C18:2n6c	4.6	16.45	16.17
C18:3 n-6	0.11	0.05	0.06
C20:2 n-6	0.09	0.08	0.08
C20:3 n-6	0.12	0.05	0.05
C20:4 n-6	1.02	0.46	0.44
n-6*Σ*PUFA	6.02	17.13	16.84
C18:3 n-3	0.64	1.36	1.37
C20:5 n-3(EPA)	7.33	0.41	0.42
C22:6 n-3(DHA)	10.48	0.04	0.05
n-3*Σ*PUFA	18.46	1.81	1.84

Abbreviations: SFA: saturated fatty acids; MUFA: monounsaturated fatty acids; n-6PUFA: n-6 polyunsaturated fatty acids; n-3PUFA: n-3 polyunsaturated fatty acids.

**Table 3:** Fatty acid composition of three experimental diets (% total fatty acids).

Fatty acid	FM	DF	FF
C12:0	0.08	3.69	8.31
C14:0	5.4	5.03	4.74
C15:0	0.49	0.39	0.25
C16:0	22.02	22.52	23.09
C17:0	0.82	0.68	0.5
C18:0	5.45	5.22	4.61
C20:0	0.75	0.56	0.22
C21:0	0.11	0.08	0.06
C22:0	0.22	0.17	0.07
C23:0	0.5	0.39	0.23
C24:0	1.9	1.52	1.08
*Σ*SFA	37.73	40.25	43.16
C15:1	0.22	0.17	0.16
C16:1	6.5	5.6	4.63
C17:1	1.22	0.99	0.46
C18:1trans	0.21	0.2	22.61
C18:1CIs	15.47	18.38	2.13
C22:1	0.18	0.12	0.06
*Σ*MUFA	23.8	25.46	30.04
C18:2n6t	0.03	0.06	0.03
C18:2n6c	13.52	14.76	14.15
C18:3 n-6	0.16	0.14	0.12
C20:2 n-6	0.14	0.12	0.10
C20:3 n-6	0.14	0.12	0.11
C20:4 n-6	0.88	0.79	0.72
n-6*Σ*PUFA	14.88	15.99	15.23
C18:3 n-3	1.64	1.59	1.24
C20:5 n-3(EPA)	10.21	7.86	5.15
C22:6 n-3(DHA)	9.68	7.21	4.08
n-3*Σ*PUFA	21.53	16.65	10.47

Abbreviations: SFA: saturated fatty acids; MUFA: monounsaturated fatty acids; n-6PUFA: n-6 polyunsaturated fatty acids; n-3PUFA: n-3 polyunsaturated fatty acids; EPA: eicosapentaenoic acid; DHA: docosahexaenoic acid.

**Table 4:** Primers used in quantitative real-time PCR.

Target genes	Sequences of primers (5′-3′)	GenBank number
*Claudin-like*	F: ATGTGGAGGGTGTCTGCC	KU238181
	R: CTGGAGGTCGCCACTGAG	
*Claudin-3*	F: GCCAGATGCAGTGTAAGGTC	KU238180
	R: CCGTCCAGGAGACAGGGAT	
*JAM*	F: CCAAGATGGACACCGGAACT	MT787206
	R: CCTCCGGTGTTTAGGTCACG	
*Tricellulin*	F: GCCTACATCCACAAAGACAACG	KU238183.1
	R: TCATTCCCAGCACTAATACAATCAC	
*ZO-1*	F: CGCCACCAGCAAAACCAGTC	AY008305.1
	R: CGATGAAGATGCCCACGTCG	
*IL-1β*	F: CGCTTCCCCAACTGGTACAT	AJ295836.2
	R: ACCTTCCACTTTGGGTCGTC	
*TNF-α*	F: GGACAGGGCTGGTACAACAC	AJ276709.1
	R: TTCAATTAGTGCCACGACAAAGAG	
*TGF-β*	F: ATGATCGACGACGAAGGCTC	KU238187.1
	R: TGGCTTTGTAGACCTCTGCG	
*β-Actin*	F: CAGGCACCAGGGAGTGATG	AY008305.1
	R: ACAATACCGTGCTCGATGGG	

**Table 5:** The growth performance of turbot.

Group	FM	DF	FF
IBW (g)	12.64 ± 0.02	12.63 ± 0.04	12.66 ± 0.04
FBW (g)	70.09 ± 0.23^a^	64.48 ± 3.11^ab^	58.05 ± 1.57^b^
SGR (%/day)	2.21 ± 0.00^a^	2.10 ± 0.06^ab^	1.96 ± 0.04^b^
WGR (%)	454.44 ± 2.09^a^	410.55 ± 22.96^ab^	358.68 ± 13.67^b^
FE	1.33 ± 0.02^a^	1.27 ± 0.03^ab^	1.21 ± 0.03^b^
FI (%/day)	1.33 ± 0.01	1.38 ± 0.02	1.38 ± 0.04
SR (%)	100 ± 0.00	99.17 ± 0.83	100 ± 0.00

The letters with different superscripts in the same row mean (*n* = 3) the difference was significant (*P* < 0.05).

**Table 6:** Alpha diversity index of the intestinal microbiota of turbot.

Group	Richness estimates			Diversity estimates	
	Observed species	chao1	Ace	Simpson	Shannon
FM	533.00 ± 127.00	551.00 ± 126.00	559.00 ± 126.00	0.79 ± 0.05	4.41 ± 0.53
DF	441.00 ± 45.00	479.00 ± 51.00	494.00 ± 54.00	0.66 ± 0.09	3.34 ± 0.54
FF	378.00 ± 44.00	415.00 ± 46.00	428.00 ± 46.00	0.83 ± 0.06	4.16 ± 0.50

The letters with different superscripts in the same row mean (*n* = 6) the difference was significant (*P* < 0.05).

## Data Availability

Raw data supporting the conclusions of this manuscript will be made available by the authors, without undue reservation, to any qualified researcher.

## Supplementary Materials

Supplementary Materials Table S1: amino acid composition of three raw materials (% dry matter basis). Table S2: amino acid composition of three experimental diets (% dry matter basis).
