# Supplementary material for: Fishmeal Protein Replacement by Defatted and Full-Fat Black Soldier Fly Larvae Meal in Juvenile Turbot Diet: Effects on the Growth Performance and Intestinal Microbiota
Source: Aquac Nutr. 2023 Apr 12;2023:8128141. doi: 10.1155/2023/8128141 (PMC10115534; doi:10.1155/2023/8128141)
Supplement: Supplementary Materials — Table S1: amino acid composition of three raw materials (% dry matter basis). Table S2: amino acid composition of three experimental diets (% dry matter basis). [file 8128141.f1.docx]

**SUPPLEMENTARY INFORMATION**

**Fishmeal protein replacement by defatted and full-fat black soldier fly larvae meal in juvenile turbot diet: Effects on the growth performance and intestinal microbiota**

Jingjing Zhao^a^, Jintao Pan^a^, Zhonghao Zhang^a^, Zhichu Chen^a^, Kangsen Mai^ab^, Yanjiao Zhang^ab*^

*^a^ Key Laboratory of Aquaculture Nutrition and Feed, Ministry of Agriculture, The Key Laboratory of Mariculture, Ministry of Education, Ocean University of China, 5 Yushan Road, Qingdao 266003, China*

*^b^ Qingdao National Laboratory for Marine Science and Technology, 1 Wenhai Road, Qingdao 266237, China*

**Corresponding author:**

Yanjiao Zhang, Ocean University of China, Qingdao 266003, China. Tel./fax: +86-532-82031627. E-mail: yanjiaozhang@ouc.edu.cn

**Table S1 Amino acids composition of three raw material (% dry matter basis).**

| Components | fish meal | defatted BSFL | full-fat BSFL |
| --- | --- | --- | --- |
| EAA |  |  |  |
| Arginine | 3.65 | 1.73 | 1.19 |
| Histidine | 1.06 | 1.02 | 0.73 |
| Isoleucine | 2.87 | 1.62 | 1.13 |
| Leucine | 4.64 | 2.48 | 1.73 |
| Lysine | 3.35 | 2.39 | 1.87 |
| Methionine | 1.19 | 1.93 | 1.43 |
| Phenylalanine | 3.28 | 1.59 | 1.13 |
| Threonine | 2.66 | 1.45 | 1.02 |
| Valine | 3.24 | 2.06 | 1.45 |
| NEAA |  |  |  |
| Alanine | 5.00 | 2.28 | 1.59 |
| Aspartic acid | 4.93 | 3.25 | 2.32 |
| Cysteine | 0.77 | 0.00 | 0.00 |
| Glutamate | 6.80 | 4.04 | 2.88 |
| Glycine | 2.64 | 2.12 | 1.47 |
| Proline | 2.36 | 2.27 | 1.31 |
| Serine | 3.85 | 1.43 | 1.02 |
| Tyrosine | 1.94 | 2.09 | 1.42 |

Abbreviations: EAA: essential amino acid; NEAA: non-essential amino acid.

**Table S2 Amino acids composition of three experimental diets (% dry matter basis)**

| Components | FM | DF | FF |
| --- | --- | --- | --- |
| EAA |  |  |  |
| Arginine | 2.23 | 2.39 | 2.19 |
| Histidine | 1.32 | 1.39 | 1.29 |
| Isoleucine | 1.9 | 2.11 | 1.93 |
| Leucine | 3.67 | 3.95 | 3.63 |
| Lysine | 2.82 | 3.03 | 2.78 |
| Methionine | 1.07 | 1.25 | 1.14 |
| Phenylalanine | 2.13 | 2.25 | 2.04 |
| Threonine | 1.72 | 1.86 | 1.72 |
| Valine | 1.91 | 2.10 | 1.92 |
| NEAA |  |  |  |
| Alanine | 2.78 | 2.98 | 2.72 |
| Aspartic acid | 3.67 | 4.02 | 3.72 |
| Cysteine | 0.39 | 0.44 | 0.45 |
| Glutamate | 6.46 | 6.92 | 6.40 |
| Glycine | 2.19 | 2.42 | 2.21 |
| Proline | 1.86 | 2.03 | 3.23 |
| Serine | 1.7 | 1.86 | 1.70 |
| Tyrosine | 1.45 | 1.71 | 1.52 |

Abbreviations: EAA: essential amino acid; NEAA: non-essential amino acid.
